# Curcumin Confers Anti-Inflammatory Effects in Adults Who Recovered from COVID-19 and Were Subsequently Vaccinated: A Randomized Controlled Trial

**DOI:** 10.3390/nu15071548

**Published:** 2023-03-23

**Authors:** Samantha N. Fessler, Yung Chang, Li Liu, Carol S. Johnston

**Affiliations:** 1 College of Health Solutions, Arizona State University, Phoenix, AZ 85004, USA; sfessler@asu.edu (S.N.F.);; 2 Biodesign Institute, Arizona State University, Tempe, AZ 85281, USA; 3 School of Life Sciences, Arizona State University, Tempe, AZ 85281, USA

**Keywords:** curcumin, COVID-19, SARS-CoV-2, inflammation, dietary supplements, cytokines

## Abstract

COVID-19 infection and vaccination offer disparate levels of defense against reinfection and breakthrough infection. This study was designed to examine the effects of curcumin supplementation, specifically HydroCurc (CURC), versus placebo (CON) on circulating inflammatory biomarkers in adults who had previously been diagnosed with COVID-19 and subsequently received a primary series of monovalent vaccine doses. This study was conducted between June 2021 and May 2022. Participants were randomized to receive CURC (500 mg) or CON capsules twice daily for four weeks. Blood sampling was completed at baseline and week-4 and analyzed for biomarkers. Linear regression was utilized to examine the between-group differences in post-trial inflammatory biomarker levels, adjusting for baseline and covariates including age, sex, race/ethnicity, and interval between COVID-19 diagnosis and trial enrollment. The sample (*n* = 31) was 71% female (Age 27.6 ± 10.4 y). The CURC group exhibited significantly lower post-trial concentrations of proinflammatory IL-6 (*β* = −0.52, 95%CI: −1.03, −0.014, *p* = 0.046) and MCP-1 (*β* = −0.12, 95%CI: −0.23, −0.015, *p* = 0.027) compared to CON, adjusting for baseline and covariates. Curcumin intake confers anti-inflammatory activity and may be a promising prophylactic nutraceutical strategy for COVID-19. These results suggest that 4 weeks of curcumin supplementation resulted in significantly lower concentrations of proinflammatory cytokines in adults who recovered from COVID-19 infection and were subsequently vaccinated.

## 1. Introduction

SARS-CoV-2 is a positive-sense single-stranded RNA coronavirus which causes the respiratory illness COVID-19, first identified in December 2019 in Wuhan, China. Since 2019, over 750 million individuals worldwide have been diagnosed with COVID-19, with infections ranging from asymptomatic to critical illness [1]. Further, COVID-19 symptoms can present in a heterogeneous manner and vary over the course of infection with the potential to impact multiple organ systems. The COVID-19 pandemic has placed historic burdens on the healthcare and economic systems in the US. To date COVID-19 is responsible for more than 1 million deaths in the US and is attributed to over 20.5 million years of life lost worldwide [2,3]. Though abundant evidence supports the efficacy of vaccines to reduce COVID-19 infections, vaccine effectiveness may wane over time with emergence of new variants and there is evidence that vaccine breakthrough infection and reinfection rates are rising [4,5,6,7]. Additionally, while vaccinations provide robust protection from severe infection, vaccinated COVID-19 cases may still spread the virus to others [8,9]. Therefore, vaccinations and updated booster doses remain central to reducing the spread and mortality related to COVID-19, yet do not eliminate the risks for severe illness and transmission. 

Importantly, the presence of elevated inflammation has been demonstrated to impact COVID-19 outcomes and prolonged inflammation and viral persistence have been demonstrated in convalescent patients [10,11]. Furthermore, aging and conditions such as obesity that are characterized by chronic inflammation and immune dysregulation have been associated with more severe disease course and post-acute COVID-19 sequalae [12,13,14]. Taken together, the widespread and devastating consequences of the COVID-19 pandemic have highlighted the importance of a well-functioning immune system, and thus an immune response that is proportionate and protective against severe illness following exposure to SARS-CoV-2 and emerging variants. Importantly, dietary components play critical roles in regulating metabolism and immune function, which are two inextricably linked processes that support the host response to pathogens [15]. Thus, safe nutritional strategies with known anti-inflammatory properties may provide complimentary prophylactic benefits to enhance immune responses with few adverse consequences.

In this context, curcumin, a polyphenolic compound derived from the rhizomes of turmeric (*Curcuma Longa*), providing the spices’ yellow pigment, has been recognized for its anti-inflammatory, antioxidant, anti-viral, and immune modulating properties [16,17]. Several studies have demonstrated the effect of curcumin to inhibit inflammatory signaling pathways such as nuclear factor-kappa B (NF-kB) in several cell types and disease models and reduce the induction of several proinflammatory cytokines and chemokines including interleukin (IL)-6, interferon (IFN) γ, monocyte chemoattractant protein (MCP)-1, tumor necrosis factor (TNF)-α, and IL-1β [18,19,20].

However, the attempts to translate findings from mechanistic studies of curcumin in clinical trials examining its effects on inflammation in healthy individuals and those with inflammatory diseases have yielded inconsistent results [21,22,23]. Importantly, this may be due to the described low bioavailability of traditional forms of curcumin and turmeric related to the poor solubility, instability, and rapid hepatic metabolism and elimination of curcumin in humans [24]. Therefore, clinical trials which aim to assess the effects of curcumin formulations with demonstrated enhancements to bioavailability are needed to understand the effects of curcumin on circulating markers of inflammation. Furthermore, given the evidence to suggest the anti-viral and anti-inflammatory activity of curcumin, the interest in this dietary compound as an adjunctive strategy for COVID-19 has risen. Importantly, curcumin has also exhibited good safety and tolerability profiles in clinical trials, even at high doses [25]. 

Given the immunomodulatory mechanisms of curcumin and the rise in breakthrough infections coinciding with the emergence SARS-CoV-2 variants, curcumin may offer anti-inflammatory and prophylactic benefits in healthy individuals with a history of prior COVID-19 infection who received the COVID-19 vaccine. In this context, the research aimed to contribute to the literature related to curcumin in a novel manner, and to examine the effects of four weeks of curcumin supplementation on circulating mediators of inflammation in healthy adults who previously tested positive for COVID-19 and subsequently received a primary series of monovalent vaccine doses, compared to a placebo control. Further, we utilized a novel formulation of curcumin, specifically HydroCurc, which has been shown to significantly increase the absorption of curcumin compared to a standard form [26]. 

## 2. Materials and Methods

### 2.1. Participants

This study was conducted between June 2021 and May 2022. Healthy adults between the ages of 18 and 65 years, who had previously tested positive for COVID-19 as per a positive RT-PCR test, and had subsequently received the full primary series of monovalent vaccine doses were recruited via online advertisements, social media, existing list-serves, and word of mouth to participate in this study. Participants were eligible if they previously experienced asymptomatic, mild, or moderate COVID-19 infection, as defined by the National Institutes of Health (NIH) COVID-19 Treatment Guidelines, and subsequently received the full primary series of a COVID-19 vaccine at least 3 months prior to enrollment [27]. Additionally, those who had received a monovalent booster dose were included in the study if this dose was administered >3 months prior to participation. Exclusion criteria included the presence of any unstable or serious illness or mood disorder, neurological conditions such as multiple sclerosis, or cognitive damage, those who received cancer treatment in the previous 5 years, pregnant or lactating females, smoking, nicotine, or drug use, alcohol use (reported at >14 alcoholic beverages per week), those who reported allergies to any ingredients in the treatment or placebo formulas, prescription of drugs known to affect immune responses or steroids, and a body mass index (BMI) > 40 kg/m^2^. Interested and eligible participants received an electronic consent form and were scheduled to visit the test site. Study visits were conducted in accordance with Centers for Disease Control and Prevention (CDC) procedures for workplace safety. The Arizona State University Institutional Review Board approved this study (STUDY 00012406), and all participants provided written consent. The study is registered at ClinicalTrials.gov, accessed on 1 March 2023, (NCT04912921).

### 2.2. Study Protocol

Initially this study was designed to recruit participants who has recently tested positive for COVID-19 (within the prior 10–15 days) and were unvaccinated. However, in December of 2020, the Pfizer-BioNTech COVID-19 vaccine was made available under emergency use authorization by the US Food and Drug Administration (FDA) [28]. Given the prioritization and protection of vaccinations, we amended our study protocol to accommodate the increase in vaccinations in the Phoenix metro area, and to ensure our research did not become a barrier to those eligible for vaccination. It is also pertinent to note that bivalent vaccine boosters did not become available until September 2022, after this study was complete [29]. The protocol of this pilot study, as described here, reflects this alternation to our study design which was approved by the Institutional Review Board at Arizona State University.

This study followed a randomized placebo-controlled parallel arm trial design. Participants were randomized by coin toss to receive the active ingredient dietary supplement or placebo for four weeks. Randomization was carried out by a blinded research team member who was not involved in data collection. Participants interested in the study were initially screened for eligibility via an online survey. Those who met inclusion criteria per the online screener were interviewed by phone to confirm eligibility. Eligible participants were scheduled to visit the test site (ISTB8 Phoenix Biomedical Collaborative Building; Phoenix, AZ, USA). 

The study participants visited the test site twice during the study period, at baseline and week 4. Study measures conducted during the baseline visit were repeated at week 4. During study visits, participants completed a standard health history questionnaire and were led through a multiple pass 24-h dietary recall by trained study staff. Study staff blinded to participant group assignment analyzed data from diet recalls using The Food Processor^®^ Nutrition and Fitness Software from ESHA Research, Inc. (Version 11.4, ©2018). Additionally, anthropometric measures were collected including height, weight, and BMI (kg/m^2^). During study visits, venous blood samples were collected via standard phlebotomy techniques into appropriate vacutainers for subsequent processing and analyses of circulating inflammatory biomarkers. At baseline, all study participants were provided with a calendar to track supplement ingestion and received directions on supplement intake and COVID-19/adverse symptom reporting in written and email forms. After four weeks of treatment or placebo ingestion, participants returned to the test site for the final study visit in which baseline assessments and blood sampling were repeated. Participants were also directed to return any unemptied supplement bottles at the final visit for adherence tracking.

### 2.3. Dietary Supplementation

Participants assigned to the active treatment group (CURC) ingested 500 mg HydroCurc^®^ twice a day for the four-week trial period. Each 500 mg dose of HydroCurc contains over 90% curcuminoids complexed with LipiSperse. LipiSperse is a novel delivery system shown to enhance the bioavailability of hydrophobic molecules. In a 2019 study by Briskey et al., the formulation of curcumin with Lipisperse demonstrated enhanced bioavailability with significantly increased plasma curcuminoids as compared to a standard curcumin formulation in healthy adults [26]. This study demonstrated a single delivered dose of 800 mg curcumin (90% curcuma longa extract, 10% Lipisperse) increased absorption compared to standard curcumin and resulted in a three-fold increase in peak plasma concentration (Cmax), which was significantly greater than Cmax for standard curcumin group [26]. Curcuminoids have been approved by the FDA as “generally recognized as safe” (GRAS), with good tolerability and safety profiles shown in previous clinical trials, even at doses up to 8 g/day [25,30]. Those in the placebo group (CON) consumed placebo capsules (maltodextrin) twice a day for four weeks. CURC and CON supplements were identical in appearance and consumed in equal capsule amounts. The supplement blinding was completed by a study team member who did not engage in data collection or analyses. At week 4 of the intervention, unemptied pill bottles were returned, and participant adherence was determined by pill count. Participants were also directed to record daily ingestion on the calendar provided to them at the start of the trial. Further, all study participants received weekly emails from investigators that contained reminders related to the study protocol.

### 2.4. Analysis of Blood Samples

At baseline and week 4, participant blood samples were collected from the antecubital vein via standard phlebotomy techniques into appropriate vacutainers. Blood was collected in 4 mL dipotassium EDTA tubes and 8.5 mL serum separator tubes. After centrifugation, a portion of collected serum was aliquoted and stored at −80 °C until analyses of cytokines and adhesion molecules. Whole blood was transported and analyzed by Sonora Quest Laboratories (Arizona) for complete blood count with differentials, which was utilized for computation of neutrophil/lymphocyte ratios (NLR). Serum was also analyzed for high sensitivity C-reactive protein (hsCRP) and ferritin by Sonora Quest Laboratories. Soluble P-selectin (sP-selectin) and soluble intercellular adhesion molecule 1 (sICAM-1) levels in serum samples were analyzed via ELISA methods (Invitrogen, Thermo Fisher Scientific, Waltham, MA, USA). Circulating serum cytokines and chemokines were analyzed via human-focused 15-plex discovery assay (Eve Technologies Corporation, Calgary, Canada). This biomarker panel provided quantitation of IL-6, IL1-β, IL-1receptor agonist (Ra), IL-2, IL-4, IL-5, IL-8, IL-10, IL-12p40, IL-12p70, IL-13, MCP-1, IFN-γ, granulocyte-macrophage colony-stimulating factor (GM-CSF), and TNF-α. 

### 2.5. Statistical Analysis

Demographic and baseline participant characteristic data are displayed as the mean ± SD or number (%). We aimed to investigate the effects of curcumin supplementation for four weeks on a panel of circulating cytokines compared to the placebo. Initially, we examined histograms of cytokine concentrations and applied log transformations to reduce skewness prior to further analyses. Additionally, cytokine concentrations below the limit of assay detection were replaced with the lowest detected value of the marker divided by 5. A series of linear regression models was used to examine the between-group differences in post-trial biomarker concentrations, controlling for baseline and covariates including age, sex, race/ethnicity, and interval between COVID-19 diagnosis and study enrollment. A nominal *p* value *<* 0.05 indicated statistical significance. Given the exploratory nature of this study, we did not adjust the outcomes for multiple comparisons, and the interpretation of the results should be considered with this in mind. Data were analyzed using open-source R software.

## 3. Results

### 3.1. Study Participants and Intervention Compliance

**Figure 1:**
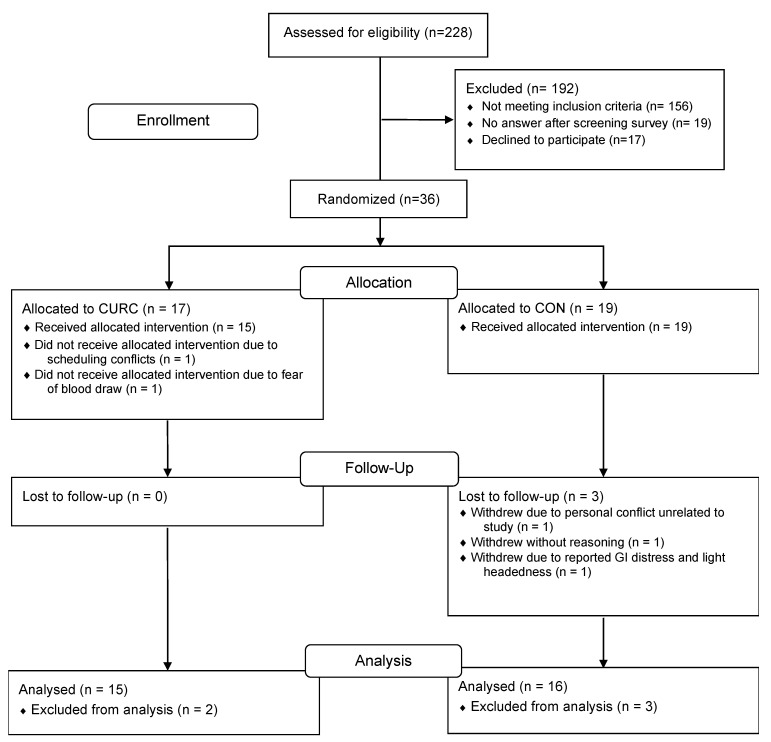
CONSORT flow diagram of study participants.

We received 288 respondents to the initial screening survey. Of the participants screened for eligibility, a total of 36 participants met inclusion criteria and were enrolled in the trial. One participant randomized to the CURC group withdrew prior to the study initiation due to scheduling conflicts, and one participant withdrew prior to blood sampling due to fear of phlebotomy. All participants randomized to the CON group received the allocated treatment. However, three participants in the CON group were lost to follow-up. One participant withdrew due to adverse reporting of gastrointestinal distress and light-headedness, one withdrew due to personal conflicts unrelated to the study, and one participant withdrew for undisclosed reasons. Therefore, 31 participants (*n* = 15 in CURC, *n* = 16 in CON) completed the trial (Figure 1) and were included in the analyses. Compliance to supplement ingestion was greater than 80% in both groups based on returned supplement bottle counts. Baseline characteristics of participants are displayed in Table 1. The sample was 71% female and the mean interval between COVID-19 diagnosis and trial enrollment was 277.7 ± 109.1 days across all participants. Participant characteristics at baseline were comparable between CURC and CON groups. Additionally, of the 31 participants who completed the trial, the types of vaccines received were similar between groups. Importantly, no participants reported a new COVID-19 diagnosis or onset of COVID-19-associated symptoms during the trial period.

### 3.2. Dietary Intakes

Dietary analysis was completed based on structured 24 h dietary recalls performed by a trained study staff member following the multiple pass method at baseline and week 4. Analysis of diet records obtained throughout the trial did not reveal significant differences between the CURC and CON groups based on the change (post-pre) in dietary intakes of energy, carbohydrates, proteins, fats, saturated fatty acids, sugar, calcium, iron, sodium, or potassium (Supplementary Table S1).

### 3.3. Inflammatory Biomarkers

We aimed to examine the between group differences in post-trial inflammatory biomarker concentrations adjusting for baseline levels of the outcome and covariates. The results of the linear regression analyses revealed that assignment to the CURC group was associated with significantly lower mean post-trial concentrations of the inflammatory biomarkers, IL-6 (*β* = −0.52, 95% CI: −1.03,−0.014, *p* = 0.046) and MCP-1 (*β* = −0.12, 95% CI: −0.23, −0.015, *p* = 0.027) controlling for baseline and covariates, compared to the CON group (Table 2 and Table S2). Additionally, while mean hsCRP at post-trial was also lower in the CURC group compared to CON group, this was not statistically significant (*p* = 0.08). There were no significant differences between groups in other circulating inflammatory markers examined (Table 2). These results suggest that curcumin supplementation for four weeks mitigated the elevations in IL-6 and MCP-1 that were observed in the CON group.

## 4. Discussion

In this double-blinded randomized placebo-controlled parallel arm trial, we demonstrated the anti-inflammatory effects of ingesting 500 mg of HydroCurc twice daily on circulating inflammatory biomarkers in adults who had previously tested positive for COVID-19 and were subsequently vaccinated, compared to placebo. Specifically, after four weeks of supplementation, the CURC group had significantly lower mean concentrations of circulating IL-6 (*p* = 0.046) and MCP-1 (*p* = 0.027), with baseline and covariate adjustment. Importantly, both IL-6 and MCP-1 are considered pro-inflammatory and prognostic of persistent and acute inflammatory dysregulation in chronic conditions and infectious diseases such as COVID-19. As per review of the literature, this is the first study to examine the anti-inflammatory and prophylactic benefits of curcumin in individuals with previous COVID-19 infection and subsequent vaccination. 

Curcumin ((1E,6E)-1,7-bis(4-hydroxy-3-methoxyphenyl)-1,6-heptadiene-3,5-dione) is the primary bioactive polyphenolic compound derived from turmeric, which has been used in the context of traditional Asian medicine for its anti-inflammatory, anti-viral, and anti-oxidative properties [31]. Therefore, the use of curcumin in various disease contexts has been widely investigated. Mechanistic studies have demonstrated that curcumin mitigates the viral binding and entry into host cells and reduces infectivity of enveloped viruses [32,33]. Additionally, recent in silico studies have suggested that curcumin has a high affinity for the SARS-CoV-2 viral S-protein and human angiotensin-converting enzyme 2 (ACE2) and may therefore interfere with SARS-CoV-2 viral entry [34]. Further, there is abundant evidence to suggest that curcumin attenuates inflammation, which plays a critical role in the development of the severe COVID-19 demonstrated in individuals with chronic inflammatory conditions [35]. Additionally, recent studies suggest that dysregulated inflammatory responses may persist after the resolution of acute COVID-19 [10]. Therefore, strategies that reduce inflammation may confer benefits even after the resolution of acute infection.

In this present study, key indicators of inflammation (IL-6 and MCP-1) rose significantly in the control group over the course of the 4-week study in comparison to the curcumin supplemented group. These data suggest that curcumin supplementation may help to manage inflammation and promote resilience. MCP-1 is a chemokine produced in response to inflammatory stimuli which promotes monocyte chemotaxis and modulates leukocyte trafficking, further propagating the inflammatory response [36,37]. The production of MCP-1 in several cell types is upregulated by signaling pathways including NF-κB and mitogen-activated protein kinase (MAPK) [38,39]. IL-6 is a proinflammatory cytokine which plays a critical role in chronic inflammation and the cytokine storm characteristic of severe COVID-19 [40]. IL-6 exerts inflammatory effects through the activation of signal transducer and activator of transcription 3 (STAT3), which amplifies NF-κB activity and further propagates the production of proinflammatory cytokines including IL-6 [41]. This positive feedback loop of IL-6 activity can be initiated by a variety of stimuli including infection, obesity, and stressors further promoting inflammation. Further, IL-6 has been shown to exhibit prothrombotic effects [42]. Importantly IL-6 and MCP-1 are upregulated in inflammatory diseases and have been associated with disease progression and severity of COVID-19 infection [43]. Therefore, aberrant and persistent elevations in IL-6 and MCP-1 may be associated with the risk for acute and chronic inflammatory conditions.

Curcumin has been demonstrated to suppress the expression of inflammatory cytokines, including IL-6 and MCP-1, from lung tissue in a murine model of virus-induced acute respiratory distress syndrome (ARDS), potentially through a reduction in NF-κB activation [19]. Additionally, a recent study demonstrated that pulmonary administration of water-soluble curcumin reduced the production of proinflammatory cytokines, such as IL-6, in the serum of mice with Klebsiella-induced pneumonia [20]. There is also evidence to suggest that curcumin reduces oxidized-LDL induced MCP-1 production in rat vascular smooth muscle cells via suppression of the p38 MAPK and NF-κB pathways [44]. Moreover, curcumin has been shown to the inhibit activation of pattern recognition receptors, such as Toll-like receptor 4 (TLR4), which play important roles in regulating the inflammatory response in the presence of infection via activation of cellular signaling pathways such as NF-κB [45]. Research has shown that curcumin modulates NF-κB activity in several cell types and animal models [46,47,48]. Additionally, curcumin treatment has been shown to mitigate inflammation through the inhibition of the NLR family pyrin domain containing 3 (NLRP3) inflammasome in murine bone-marrow-derived macrophages and the activation of production of cyclooxygenase 2 (COX-2) in murine models of chronic obstructive pulmonary disease (COPD), an enzyme associated which the biosynthesis of proinflammatory eicosanoid synthesis [49,50].

Importantly, there is promising evidence from clinical trials to support the anti-inflammatory role of curcumin in adults with varying levels of health. A recent meta-analysis examining the effects of curcumin on circulating IL-6 in various adult populations from nine clinical trials, found that supplementation significantly reduced IL-6 concentrations [51]. Another recent systematic review and meta-analysis which investigated the antioxidant and anti-inflammatory of curcumin from sixty-six randomized controlled trials found that curcumin significantly lowered the levels of CRP, TNF-α, and IL-6 [22]. However, some investigations have reported conflicting results. Inconsistent results in the literature may be related to the low bioavailability of curcumin, which exhibits poor absorption, and undergoes rapid metabolism and elimination in humans [52,53]. Therefore, the results of this study are promising as we utilized a novel curcumin formulation, HydroCurc, which has demonstrated increased bioavailability compared to standard curcumin in healthy adults. HyrdoCurc is complexed with Lipisperse technology and is suggested to enhance bioavailability via its ability to reduce curcumin agglomeration and increase curcumin absorption [26]. 

The present study is not without limitations, such as the small sample size (*n* = 31). Additionally, given the exploratory nature of this study, outcome data were not adjusted for multiple comparisons, and results should be interpreted with this consideration in mind. Finally, it is a limitation of this trial that circulating concentrations of curcuminoids were not quantified; however, a recent report which examined the bioavailability of HydroCurc, which is complexed with the Lipisperse delivery system, in healthy adults found significantly elevated curcuminoids in circulation compared to standard curcumin formulation [26].

COVID-19 has placed significant economic and societal burdens on the global population. Upon infection with SARS-CoV-2, the presentation of COVID-19 symptoms spans mild cold-like manifestations to pneumonia and severe acute respiratory distress syndrome, with the severity of disease being associated with the dysregulated release of cytokines [54]. Further, while vaccinations have been demonstrated to provide protection from severe COVID-19 outcomes, the incidence of breakthrough infections is rising, and vaccine effectiveness seems to wane over time compounded by the emergence of SARS-CoV-2 variants. It is well-documented that dietary factors are key players in promoting an optimal immune response [55]. Recently, curcumin has been postulated as a promising and safe prophylactic strategy for COVID-19. The current study provides evidence that supplementation with HydroCurc improves inflammatory profiles, specifically levels of IL-6 and MCP-1, in adults who had previously tested positive for COVID-19 and were subsequently vaccinated. Therefore, the immunomodulatory effects of curcumin may confer benefits to adults exposed to a variety of daily stressors and pathogens, including SARS-CoV-2. However, longer prospective trials are necessary to progress the findings of this study and examine if the anti-inflammatory effects of curcumin may reduce the incidence and severity of COVID-19 breakthrough infections.

## 5. Conclusions

This study investigated the anti-inflammatory and prophylactic effects of curcumin in individuals who had recovered from COVID-19 infection and were subsequently vaccinated. We demonstrated that curcumin supplementation was associated with significantly lower levels of the proinflammatory cytokines IL-6 and MCP-1, which increased in the control group during the four-week trial period. These findings indicate that curcumin supplementation may help to control inflammation and support resilience. 

## Figures and Tables

**Table 1:** Baseline characteristics of study participants (*n* = 31) in the CURC and CON groups.

	CON		CURC	
n	16		15	
**Demographics**				
Sex, Female n (%)	12	(75.0)	10	(66.7)
Age, years	27.8	±11.6	27.5	±9.5
**Anthropometrics**				
BMI, kg/m^2^	25.8	±4.9	24.8	±4.7
**Race/Ethnicity**				
White	12	(75.0)	8	(53.3)
Asian	3	(18.8)	3	(20.0)
Hispanic/Latin(o/a/x)	1	(6.3)	4	(26.7)
**COVID-19 Illness Classification**				
Asymptomatic	2	(12.5)	1	(6.7)
Mild	14	(87.5)	13	(86.7)
Moderate	0	(0)	1	(6.7)
**Days from COVID-19 Diagnosis**				
Days	252.2	±76.5	305	±133
**COVID-19 Vaccine Received**				
Pfizer-BioNTech	9	(56.3)	8	(53.3)
Moderna	4	(25.0)	4	(26.7)
Johnson & Johnson’s Janssen	3	(18.8)	2	(13.3)
AstraZeneca	0	(0)	1	(6.7)
**Monovalent Booster Status**				
n (%)	1	(6.3)	2	(13.3)

Data are presented as n (percentage) of participants or mean ± SD. BMI, body mass index.

**Table 2:** Model-based effects of CURC treatment for four weeks on log-transformed biomarkers of inflammation in serum, compared to CON ^1^.

	Estimate ^2^	(95% CI)		*p* Value
hsCRP (mg/L)	−0.19	(−0.41,	0.03)	0.08
Ferritin (ng/mL)	0.023	(−0.12,	0.16)	0.74
NLR	0.10	(−0.023,	0.22)	0.11
IL-6 (pg/mL)	−0.52	(−1.03,	−0.014)	0.045
GM-CSF (pg/mL)	−0.10	(−0.86,	0.66)	0.78
IFN-γ (pg/mL)	−0.04	(−0.39,	0.31)	0.81
IL-1β (pg/mL)	−0.35	(−1.25,	0.56)	0.44
IL-1Ra (pg/mL)	−0.08	(−0.31,	0.15)	0.49
IL-2 (pg/mL)	0.02	(−0.61,	0.64)	0.96
IL-4 (pg/mL)	−0.08	(−0.35,	0.18)	0.52
IL-5 (pg/mL)	−0.10	(−0.37,	0.16)	0.43
IL-8 (pg/mL)	−0.07	(−0.20,	0.065)	0.30
IL-10 (pg/mL)	−0.39	(−0.89,	0.11)	0.12
IL-12p40 (pg/mL)	−0.16	(−0.34,	0.023)	0.08
IL-12p70 (pg/mL)	0.30	(−0.43,	1.03)	0.40
IL-13 (pg/mL)	0.05	(−0.36,	0.47)	0.80
MCP-1 (pg/mL)	−0.12	(−0.23,	−0.015)	0.027
TNF-α (pg/mL)	−0.09	(−0.32,	0.14)	0.41
sICAM-1 (ng/mL)	−0.004	(−0.040,	0.031)	0.80
sP-selectin (ng/mL)	−0.007	(−0.064,	0.049)	0.78

^1^ Data were analyzed by multiple linear regression models with the week 4 levels of the outcome as the response variable regressed on the treatment group adjusting for baseline level of the outcome and covariates including age, sex, race/ethnicity, and interval between COVID-19 diagnosis and trial enrollment. Data are presented as regression estimates and 95%CI of the estimate for the group assignment variable from each model. ^2^ Estimates represent CURC treatment effects on the between group differences at week 4 adjusting for baseline and covariates.

## Data Availability

The data presented in this study are available on request from the corresponding author.

## Supplementary Materials

The following supporting information can be downloaded at: https://www.mdpi.com/article/10.3390/nu15071548/s1. Table S1. Nutrient intakes from 24 h dietary recalls at baseline and week four for the CURC and CON groups. Table S2. Status of circulating inflammatory cytokines and chemokines in serum by treatment group at baseline and week 4.

## Abbreviations

CURC: 500 mg HydroCurc twice daily; CON, placebo; hsCRP, high sensitivity C-reactive protein; GM-CSF, granulocyte-macrophage colony-stimulating factor; sICAM-1, soluble intercellular adhesion molecule 1; IFN, interferon; IL, interleukin; MCP-1, monocyte chemoattractant protein-1; NF-κB, nuclear factor-kappa B; N/L, neutrophil/lymphocyte ratios; sP-selectin, soluble P-selectin; TNF, tumor necrosis factor; TLR-4, Toll like receptor 4; BMI, body mass index; NIH, National Institutes of Health, CDC, Centers for Disease Control and Prevention; FDA, US Food and Drug Administration; Cmax, peak plasma concentration; ACE2, angiotensin-converting enzyme 2; MAPK, mitogen-activated protein kinase; STAT3, signal transducer and activator of transcription 3; NLRP3, NLR family pyrin domain containing 3; COX-2, cyclooxygenase 2; COPD, chronic obstructive pulmonary disease.

## References

[1] WHO (2020). WHO Coronavirus Disease (COVID-19) Dashboard. https://covid19.who.int/.

[2] Centers for Disease Control and Prevention COVID-19 Data and Surveillance. https://www.cdc.gov/coronavirus/2019-ncov/covid-data/covidview/index.html.

[3] Arolas H.P.I., Acosta E., López-Casasnovas G., Lo A., Nicodemo C., Riffe T., Myrskylä M. (2021). Years of life lost to COVID-19 in 81 countries. Sci. Rep..

[4] Johnson A.G., Amin A.B., Ali A.R., Hoots B., Cadwell B.L., Arora S., Avoundjian T., Awofeso A.O., Barnes J., Bayoumi N.S. (2022). COVID-19 Incidence and Death Rates Among Unvaccinated and Fully Vaccinated Adults with and Without Booster Doses During Periods of Delta and Omicron Variant Emergence—25 U.S. Jurisdictions, April 4–December 25, 2021. Morb. Mortal. Wkly. Rep..

[5] Bergwerk M., Gonen T., Lustig Y., Amit S., Lipsitch M., Cohen C., Mandelboim M., Levin E.G., Rubin C., Indenbaum V. (2021). Covid-19 Breakthrough Infections in Vaccinated Health Care Workers. N. Engl. J. Med..

[6] Accorsi E.K., Britton A., Fleming-Dutra K.E., Smith Z.R., Shang N., Derado G., Miller J., Schrag S.J., Verani J.R. (2022). Association Between 3 Doses of mRNA COVID-19 Vaccine and Symptomatic Infection Caused by the SARS-CoV-2 Omicron and Delta Variants. JAMA.

[7] Altarawneh H.N., Chemaitelly H., Hasan M.R., Ayoub H.H., Qassim S., AlMukdad S., Coyle P., Yassine H.M., Al-Khatib H.A., Benslimane F.M. (2022). Protection against the Omicron Variant from Previous SARS-CoV-2 Infection. N. Engl. J. Med..

[8] Thompson M.G., Stenehjem E., Grannis S., Ball S.W., Naleway A.L., Ong T.C., DeSilva M.B., Natarajan K., Bozio C.H., Lewis N. (2021). Effectiveness of Covid-19 Vaccines in Ambulatory and Inpatient Care Settings. N. Engl. J. Med..

[9] Tan S.T., Kwan A.T., Rodríguez-Barraquer I., Singer B.J., Park H.J., Lewnard J.A., Sears D., Lo N.C. (2023). Infectiousness of SARS-CoV-2 breakthrough infections and reinfections during the Omicron wave. Nat. Med..

[10] Phetsouphanh C., Darley D.R., Wilson D.B., Howe A., Munier C.M.L., Patel S.K., Juno J.A., Burrell L.M., Kent S.J., Dore G.J. (2022). Immunological dysfunction persists for 8 months following initial mild-to-moderate SARS-CoV-2 infection. Nat. Immunol..

[11] Mehandru S., Merad M. (2022). Pathological sequelae of long-haul COVID. Nat. Immunol..

[12] De La Fuente M., De Castro M.N. (2012). Obesity as a Model of Premature Immunosenescence. Curr. Immunol. Rev..

[13] Agarwal S., Busse P.J. (2010). Innate and adaptive immunosenescence. Ann. Allergy, Asthma Immunol..

[14] Sudre C.H., Murray B., Varsavsky T., Graham M.S., Penfold R.S., Bowyer R.C., Pujol J.C., Klaser K., Antonelli M., Canas L.S. (2021). Attributes and predictors of long COVID. Nat. Med..

[15] Xiao N., Nie M., Pang H., Wang B., Hu J., Meng X., Li K., Ran X., Long Q., Deng H. (2021). Integrated cytokine and metabolite analysis reveals immunometabolic reprogramming in COVID-19 patients with therapeutic implications. Nat. Commun..

[16] Prasad S., Tyagi A.K., Aggarwal B.B. (2014). Recent Developments in Delivery, Bioavailability, Absorption and Metabolism of Curcumin: The Golden Pigment from Golden Spice. Cancer Res. Treat..

[17] Kunnumakkara A.B., Bordoloi D., Padmavathi G., Monisha J., Roy N.K., Prasad S., Aggarwal B.B. (2017). Curcumin, the golden nutraceutical: Multitargeting for multiple chronic diseases. Br. J. Pharmacol..

[18] Chowdhury I., Banerjee S., Driss A., Xu W., Mehrabi S., Nezhat C., Sidell N., Taylor R.N., Thompson W.E. (2019). Curcumin attenuates proangiogenic and proinflammatory factors in human eutopic endometrial stromal cells through the NF-κB signaling pathway. J. Cell. Physiol..

[19] Avasarala S., Zhang F., Liu G., Wang R., London S.D., London L. (2013). Curcumin Modulates the Inflammatory Response and Inhibits Subsequent Fibrosis in a Mouse Model of Viral-induced Acute Respiratory Distress Syndrome. PLoS ONE.

[20] Zhang B., Swamy S., Balijepalli S., Panicker S., Mooliyil J., Sherman M.A., Parkkinen J., Raghavendran K., Suresh M.V. (2019). Direct pulmonary delivery of solubilized curcumin reduces severity of lethal pneumonia. FASEB J..

[21] White C.M., Pasupuleti V., Roman Y.M., Li Y., Hernandez A.V. (2019). Oral turmeric/curcumin effects on inflammatory markers in chronic inflammatory diseases: A systematic review and meta-analysis of randomized controlled trials. Pharmacol. Res..

[22] Dehzad M.J., Ghalandari H., Nouri M., Askarpour M. (2023). Antioxidant and anti-inflammatory effects of curcumin/turmeric supplementation in adults: A GRADE-assessed systematic review and dose–response meta-analysis of randomized controlled trials. Cytokine.

[23] Naghsh N., Musazadeh V., Nikpayam O., Kavyani Z., Moridpour A.H., Golandam F., Faghfouri A.H., Ostadrahimi A. (2023). Profiling Inflammatory Biomarkers following Curcumin Supplementation: An Umbrella Meta-Analysis of Randomized Clinical Trials. Evid.-Based Complement. Altern. Med..

[24] Anand P., Kunnumakkara A.B., Newman R.A., Aggarwal B.B. (2007). Bioavailability of curcumin: Problems and promises. Mol. Pharm..

[25] Thimmulappa R.K., Mudnakudu-Nagaraju K.K., Shivamallu C., Subramaniam K., Radhakrishnan A., Bhojraj S., Kuppusamy G. (2021). Antiviral and immunomodulatory activity of curcumin: A case for prophylactic therapy for COVID-19. Heliyon.

[26] Briskey D., Sax A., Mallard A.R., Rao A. (2019). Increased bioavailability of curcumin using a novel dispersion technology system (LipiSperse®). Eur. J. Nutr..

[27] National Institutes of Health Clinical Spectrum of SARS-CoV-2 Infection. https://www.covid19treatmentguidelines.nih.gov/overview/clinical-spectrum/.

[28] Oliver S.E., Gargano J.W., Marin M., Wallace M., Curran K.G., Chamberland M., McClung N., Campos-Outcalt D., Morgan R.L., Mbaeyi S. (2020). The Advisory Committee on Immunization Practices’ Interim Recommendation for Use of Pfizer-BioNTech COVID-19 Vaccine—United States, December 2020. MMWR. Morb. Mortal. Wkly. Rep..

[29] Centers for Disease Control and Prevention Stay Up to Date on COVID-19 Vaccines. https://www.cdc.gov/coronavirus/2019-ncov/vaccines/stay-up-to-date.html?s_cid=11747:cdc%20fully%20vaccinated%20definition:sem.ga:p:RG:GM:gen:PTN:FY22.

[30] Campbell M.S., Fleenor B.S. (2018). The emerging role of curcumin for improving vascular dysfunction: A review. Crit. Rev. Food Sci. Nutr..

[31] Peng Y., Ao M., Dong B., Jiang Y., Yu L., Chen Z., Hu C., Xu R. (2021). Anti-Inflammatory Effects of Curcumin in the Inflammatory Diseases: Status, Limitations and Countermeasures. Drug Des. Dev. Ther..

[32] Mounce B.C., Cesaro T., Carrau L., Vallet T., Vignuzzi M. (2017). Curcumin inhibits Zika and chikungunya virus infection by inhibiting cell binding. Antivir. Res..

[33] Anggakusuma, Colpitts C.C., Schang L.M., Rachmawati H., Frentzen A., Pfaender S., Behrendt P., Brown R.J.P., Bankwitz D., Steinmann J. (2014). Turmeric curcumin inhibits entry of all hepatitis C virus genotypes into human liver cells. Gut.

[34] Jena A.B., Kanungo N., Nayak V., Chainy G.B.N., Dandapat J. (2021). Catechin and curcumin interact with S protein of SARS-CoV2 and ACE2 of human cell membrane: Insights from computational studies. Sci. Rep..

[35] Xu X.-Y., Meng X., Li S., Gan R.-Y., Li Y., Li H.-B. (2018). Bioactivity, Health Benefits, and Related Molecular Mechanisms of Curcumin: Current Progress, Challenges, and Perspectives. Nutrients.

[36] Ho A.W.Y., Wong C.K., Lam C.W.K. (2008). Tumor necrosis factor-α up-regulates the expression of CCL2 and adhesion molecules of human proximal tubular epithelial cells through MAPK signaling pathways. Immunobiology.

[37] Olson T.S., Ley K. (2002). Chemokines and chemokine receptors in leukocyte trafficking. Am. J. Physiol. Regul. Integr. Comp. Physiol..

[38] Tang M., Wang Y., Han S., Guo S., Xu N., Guo J. (2013). Endogenous PGE2 induces MCP-1 expression via EP4/p38 MAPK signaling in melanoma. Oncol. Lett..

[39] Ueda A., Okuda K., Ohno S., Shirai A., Igarashi T., Matsunaga K., Fukushima J., Kawamoto S., Ishigatsubo Y., Okubo T. (1994). NF-kappa B and Sp1 regulate transcription of the human monocyte chemoattractant protein-1 gene. J. Immunol..

[40] Mehta P., McAuley D.F., Brown M., Sanchez E., Tattersall R.S., Manson J.J., on behalf of theHLH Across Speciality Collaboration, UK (2020). COVID-19: Consider cytokine storm syndromes and immunosuppression. Lancet.

[41] Hirano T. (2021). IL-6 in inflammation, autoimmunity and cancer. Int. Immunol..

[42] Bester J., Pretorius E. (2016). Effects of IL-1β, IL-6 and IL-8 on erythrocytes, platelets and clot viscoelasticity. Sci. Rep..

[43] Jørgensen M.J., Holter J.C., Christensen E.E., Schjalm C., Tonby K., Pischke S.E., Jenum S., Skeie L.G., Nur S., Lind A. (2020). Increased interleukin-6 and macrophage chemoattractant protein-1 are associated with respiratory failure in COVID-19. Sci. Rep..

[44] Zhong Y., Liu T., Guo Z. (2012). Curcumin inhibits ox-LDL-induced MCP-1 expression by suppressing the p38MAPK and NF-κB pathways in rat vascular smooth muscle cells. Inflamm. Res..

[45] Dai J., Gu L., Su Y., Wang Q., Zhao Y., Chen X., Deng H., Li W., Wang G., Li K. (2018). Inhibition of curcumin on influenza A virus infection and influenzal pneumonia via oxidative stress, TLR2/4, p38/JNK MAPK and NF-κB pathways. Int. Immunopharmacol..

[46] Han S., Xu J., Guo X., Huang M. (2018). Curcumin ameliorates severe influenza pneumonia via attenuating lung injury and regulating macrophage cytokines production. Clin. Exp. Pharmacol. Physiol..

[47] Oh S.-W., Cha J.-Y., Jung J.-E., Chang B.-C., Kwon H.-J., Lee B.-R., Kim D.-Y. (2011). Curcumin attenuates allergic airway inflammation and hyper-responsiveness in mice through NF-κB inhibition. J. Ethnopharmacol..

[48] Singh S., Aggarwal B.B. (1995). Activation of transcription factor NF-κB is suppressed by curcumin (diferuloylmethane). J. Biol. Chem..

[49] Yin H., Guo Q., Li X., Tang T., Li C., Wang H., Sun Y., Feng Q., Ma C., Gao C. (2018). Curcumin Suppresses IL-1β Secretion and Prevents Inflammation through Inhibition of the NLRP3 Inflammasome. J. Immunol..

[50] Yuan J., Liu R., Ma Y., Zhang Z., Xie Z. (2018). Curcumin Attenuates Airway Inflammation and Airway Remolding by Inhibiting NF-κB Signaling and COX-2 in Cigarette Smoke-Induced COPD Mice. Inflammation.

[51] Derosa G., Maffioli P., Simental-Mendía L.E., Bo S., Sahebkar A. (2016). Effect of curcumin on circulating interleukin-6 concentrations: A systematic review and meta-analysis of randomized controlled trials. Pharmacol. Res..

[52] Ammon H.P.T., Wahl M.A. (1991). Pharmacology of *Curcuma longa*. Planta Med..

[53] Liu W., Zhai Y., Heng X., Che F.Y., Chen W., Sun D., Zhai G. (2016). Oral bioavailability of curcumin: Problems and advancements. J. Drug Target..

[54] Yang L., Xie X., Tu Z., Fu J., Xu D., Zhou Y. (2021). The signal pathways and treatment of cytokine storm in COVID-19. Signal Transduct. Target. Ther..

[55] Calder P.C. (2021). Nutrition and immunity: Lessons for COVID-19. Eur. J. Clin. Nutr..

