# Supplementary material for: Curcumin Confers Anti-Inflammatory Effects in Adults Who Recovered from COVID-19 and Were Subsequently Vaccinated: A Randomized Controlled Trial
Source: Nutrients. 2023 Mar 23;15(7):1548. doi: 10.3390/nu15071548 (PMC10096702; doi:10.3390/nu15071548)
Supplement: Supplementary file 1 [file nutrients-15-01548-s001.zip › nutrients-2299564-supplementary.pdf]

Table S1. Nutrient intakes from 24-hour Dietary Recalls at baseline and week four for the CURC and CON groups.

	Baseline				Week 4				
	CURC		CON		CURC		CON		***P*** [1]
Calories (kcal)	1861	(1393.0 , 2593.4)	1923	(1508.9 , 2137.9)	1638	(1271.7 , 2295.6)	1418	(1062.6 , 1999.7)	0.57
Protein (g)	76.6	(60.5 , 100.3)	72.9	(61.5 , 87.3)	75.6	(70.2 , 102.6)	63.8	(42.9 , 90.3)	0.52
Carbohydrate (g)	192.7	(172.3 , 235.3)	211.8	(170.6 , 239.2)	173.4	(145.0 , 256.4)	149.6	(122.6 , 238.0)	0.44
Sugar (g)	46.5	(33.2 , 72.2)	66.5	(52.0 , 99.2)	55.2	(50.6 , 71.4)	41.1	(25.8 , 60.7)	0.08
Fat (g)	78.3	(57.9 , 126.0)	82.2	(64.4 , 94.9)	70.3	(45.8 , 96.9)	55.4	(39.1 , 71.0)	0.89
Saturated Fat (g)	27.6	(13.9 , 33.3)	25.3	(15.1 , 33.8)	19.3	(9.2 , 29.8)	16.8	(11.5 , 19.7)	0.47
Cholesterol (mg)	205.4	(85.0 , 528.8)	204.6	(152.7 , 332.3)	165.4	(89.0 , 314.2)	141.6	(99.2 , 350.2)	0.87
Calcium (mg)	647.0	(516.7 , 1006.7)	825.4	(624.2 , 1121.3)	714.3	(487.8 , 1167.7)	761.2	(467.4 , 883.8)	0.44
Iron (mg)	10.5	(9.1 , 15.0)	11.7	(7.8 , 15.4)	11.7	(9.0 , 13.5)	8.9	(7.6 , 15.0)	0.54
Potassium (mg)	1259.0	(906.2 , 2708.2)	1676.5	(1418.2 , 2469.0)	2200.3	(1310.1 , 2395.4)	1670.8	(1106.9 , 2280.6)	0.36
Sodium (mg)	2897.6	(2125.1 , 4035.2)	3002.8	(2646.7 , 3785.4)	2697.8	(1878.4 , 3705.6)	2791.3	(2024.2 , 3797.9)	0.55

Data are medians (IQR). [1]*P*-value is derived from Mann Whitney U test for change data between groups. Change data calculated as post-pre values.

Table S2. Status of circulating inflammatory cytokines and chemokines in serum by treatment group at baseline and week 4

	Baseline		Week 4	
	CURC	CON	CURC	CON
Ferritin (ng/mL)	70.07 ± 47.00	68.75 ± 55.73	66.43 ± 44.27	63.50 ± 61.38
hsCRP (mg/L)	1.26 ± 1.55	1.99 ± 2.33	1.25 ± 1.52	2.56 ± 2.68
NLR	1.82 ± 0.66	1.70 ± 0.64	2.40 ± 0.99	2.06 ± 1.16
GM-CSF (pg/mL)	5.20 ± 10.88	0.45 ± 1.65	16.13 ± 36.60	7.60 ± 14.98
IFN-γ (pg/mL)	2.83 ± 3.71	1.35 ± 1.55	5.08 ± 7.57	2.64 ± 3.06
IL-1β (pg/mL)	26.62 ± 34.18	11.69 ± 19.02	22.99 ± 25.84	19.09 ± 25.77
IL-1Ra (pg/mL)	7.04 ± 5.36	4.38 ± 2.65	7.40 ± 3.92	6.82 ± 6.35
IL-2 (pg/mL)	1.09 ± 0.89	0.30 ± 0.27	1.34 ± 1.48	0.90 ± 1.67
IL-4 (pg/mL)	1.07 ± 1.13	0.59 ± 0.43	1.88 ± 3.03	1.02 ± 1.11
IL-5 (pg/mL)	2.61 ± 2.31	3.29 ± 3.40	3.57 ± 3.15	4.96 ± 6.09
IL-6 (pg/mL)	0.85 ± 0.88	0.76 ± 0.52	0.96 ± 0.84	1.22 ± 0.82
IL-8 (pg/mL)	5.12 ± 2.68	5.16 ± 3.26	6.23 ± 2.50	6.19 ± 3.77
IL-10 (pg/mL)	0.51 ± 1.30	1.89 ± 4.11	0.62 ± 1.61	2.76 ± 4.51
IL-12p40 (pg/mL)	49.02 ± 29.03	48.79 ± 18.35	44.62 ± 28.28	65.80 ± 48.22
IL-12p70 (pg/mL)	4.29 ± 5.74	1.28 ± 2.05	6.50 ± 8.06	3.99 ± 7.18
IL-13 (pg/mL)	66.65 ± 68.61	29.59 ± 21.72	97.21 ± 118.01	44.87 ± 41.25
MCP-1 (pg/mL)	204.48 ± 77.86	215.90 ± 54.60	217.05 ± 88.50	267.40 ± 157.76
TNF-α (pg/mL)	29.28 ± 24.39	18.98 ± 7.05	42.09 ± 47.95	27.46 ± 14.65
sICAM-1 (ng/mL)	383.29 ± 59.37	422.40 ± 86.53	413.49 ± 58.27	434.17 ± 77.48
sP-selectin (ng/mL)	62.17 ± 18.97	62.93 ± 18.32	60.20 ± 22.94	60.23 ± 15.70

Data are means ± SD
